# Mark-Release-Recapture of Packed and Shipped *Aedes aegypti* with *Wolbachia*: Implications for Conducting Remote Incompatible Insect Technique Programs

**DOI:** 10.4269/ajtmh.24-0262

**Published:** 2025-03-18

**Authors:** Johanna R. Ohm, Amy Lynd, Austin McGowan, Angel Cupid, Vernessa Bellot, James Q. Le, Evdoxia Kakani, Josh Livni, Jacob E. Crawford, Bradley J. White

**Affiliations:** ^1^ Verily Life Sciences LLC, South San Francisco, California;; ^2^ Green VI, Virgin Gorda, British Virgin Islands

## Abstract

Male mosquitoes containing the endosymbiont *Wolbachia* (*Wb+*) can be used as a tool to suppress wild mosquito populations through a technique termed incompatible insect technique (IIT). IIT programs reduce wild mosquitoes via incompatible matings between released males and wild females to reduce the number of viable offspring produced in the next generation. Successful programs rely on regular release of incompatible males to outcompete wild males for female mates. Past IIT programs have relied on local production of *Wb+* males to support regular releases of incompatible males. Here, we evaluated the survival and dispersal of packed and shipped *Wb+ Aedes aegypti* males in mark-release-recapture studies at a release site in the British Virgin Islands (BVI), separated by over 3,600 miles from the centralized production facility. Released mosquitoes were recaptured using BG-Sentinel 2 traps collected daily for up to 7 days after release. *Wb+* male mosquitoes packed and shipped from a centralized production facility performed similarly to males that were locally reared in the BVI in survival, dispersal, and recapture rates. Our results support the conclusion that packing and shipping live *Wb+* male mosquitoes does not impact their ability to survive and disperse in release sites and suggests that IIT mosquito control programs can feasibly be conducted nearly anywhere in the world without the need for local mosquito production facilities.

## INTRODUCTION

Mosquito-borne arboviruses have been increasing in prevalence and geographic distribution over the past two decades, with the World Health Organization estimating an 8-fold increase in the number of dengue cases from 2000 to 2019.^1^ Over 5 million dengue cases were reported in 2019 with 3.1 million cases in the Americas.^1^ Efforts to reduce dengue and other mosquito-borne diseases have historically relied on source reduction and insecticides. There is no widespread use of vaccines and/or drugs to prevent or treat arboviruses, leaving development of more effective vector control as a promising option.

Vector control tools that rely on alternatives to chemical insecticides include incompatible insect technique (IIT), which uses a naturally occurring bacteria to cause incompatible matings between wild females and trans-infected male mosquitoes. *Aedes* (*Ae.*) *aegypti*, the primary vector of dengue, is not naturally infected with *Wolbachia* (*Wb+*) bacteria but can be trans-infected via microinjection of *Wb+* from closely related species. Trans-infected male mosquitoes are incompatible with wild female mosquitoes; e.g., eggs laid by wild females after mating with *Wb+*-carrying males exhibit cytoplasmic incompatibility and do not hatch, resulting in lower numbers of viable offspring in the next generation. When reared en masse, *Wb+*-carrying males can be released to suppress wild populations by reducing the number of viable matings. This approach has been used to suppress wild populations of *Ae. aegypti* in the United States (Houston, Texas, Miami, Florida, Ponce, Puerto Rico, and Fresno, CA), Singapore, Mexico, and Australia.^2^^–^^8^ Most of these IIT programs have relied on local or regional production of mosquitoes rather than packing and shipping from a centralized facility. Packing and shipping mosquitoes internationally over long distances for a remote IIT program presents significant challenges and has not previously been evaluated in mark-release-recapture (MRR) studies.

Key to the success of IIT release programs is an understanding of the number of *Wb+* males needed to release in areas targeted for suppression. Insufficient release numbers of *Wb+* males will fail to suppress the wild population, whereas excessive numbers of released males can irritate residents and add unnecessary expenses to rearing and release operations. In previous male *Wb+* release programs, the number of males released per week to achieve suppression ranged between 210 and 2,760 males per acre.^2^^–^^8^ The target male release rate, spacing of release points, and the frequency of releases are dependent on the size of the wild population, mating competitiveness, the daily survival probability, and dispersal distance of released males.

MRR studies ahead of releases for suppression can help inform release strategies by estimating the dispersal distance, probability of daily survival (PDS), and efficiency of traps used in the release areas. Additionally, MRR studies can be used to evaluate differences in survival and dispersal between released mosquitoes using different release methods to help refine the release technique. Finally, MRR studies have the ability to estimate the population size of the local population to inform the number of *Wb+* males needed to outnumber wild male competitors in sufficient numbers (ratio of ∼10:1 suggested in Dobson 2021).^9^

Previous MRR studies have used fluorescent powders, *Wb+* infection, ingestible dyes, immunomarking, trace elements, or stable isotopes to identify recaptured mosquitoes or other flying insects.^10^^–^^18^ Each method has limitations. Trace elements and immunomarking can give false positives from environmental contaminants.^16^ Trace elements used in larval rearing inflict fitness costs and are not well retained in some species, which can bias results against marked mosquitoes.^17^ Fluorescent dusts have been found to reduce recapture rates and can impact survival.^19^^,^^20^ Newer methods such as spraying or feeding DNA oligonucleotides to “barcode” mosquitoes offer promise to avoid these limitations but require screening using molecular techniques.^21^^,^^22^ A novel application of fluorescent marking using a nebulizer and adhesive polymer to control the amount of marker applied and reduce potential fitness costs of marking has recently been combined with DNA barcodes to identify recaptured mosquitoes quickly in the field and expand the range of available markers beyond fluorescent colors alone.^21^ Recent studies in other insects have confirmed that these liquid fluorescent markers persist and can be used for MRR with good success.^23^^–^^24^ Here, we used the method to apply fluorescent visible markers presented in Faiman et al. 2021, to compare survival, dispersal, and recapture rates of packed and shipped *Wb+* male *Ae. aegypti* to locally reared wild-type (WT) males, allowing us to evaluate the fitness of packed and shipped *Wb*+ males intended for IIT releases compared with WT competitors.^21^ We were also able to compare the impact of the fluorescent marking technique to unmarked mosquitoes by using the presence of *Wb+* infection as an additional marker for *Wb+*-released males. We compared survival, dispersal distance, and recapture rates of *Wb+* males with and without fluorescence to evaluate potential costs of fluorescent marking.

We conducted a series of two MRR studies with two independent replicates (reps) in an area targeted for an IIT release program to assess whether packed and shipped *Wb+* male mosquitoes had similar survival and dispersal compared with WT mosquitoes. In the second study, we additionally evaluated whether packed and shipped mosquitoes could be released directly from shipping boxes to reduce operational complexity, compared with protocols where males are unpackaged and sugar-fed before release. The studies were conducted in the British Virgin Islands (BVI), an overseas territory of the United Kingdom. The territory has above-average dengue incidence compared with other Caribbean islands with 71 cases per 100,000 people in 2022.^25^ The territory has a population of 31,122 and consists of 50 different small islands with the second largest population on Virgin Gorda.^26^ An IIT suppression program began in Virgin Gorda in January 2023 after completion of these MRR studies. Below, we describe our MRR studies and the differences observed in survival, dispersal, and recapture between *Wb+* males, marked or unmarked with fluorescence, and marked WT males. In our second MRR, we altered release methods for packed and shipped *Wb+* males to release directly from the containers they were shipped in (“shipping pucks”) instead of unpacking and holding overnight ahead of release. We additionally conducted assays on mating competitiveness of packed and shipped *Wb+* males to confirm males were competitive with WT males in mating with WT females. We discuss implications of our results for the ongoing IIT release program in the BVI and for future IIT programs in remote locations.

## MATERIALS AND METHODS

### Study area.

**Figure 1.**
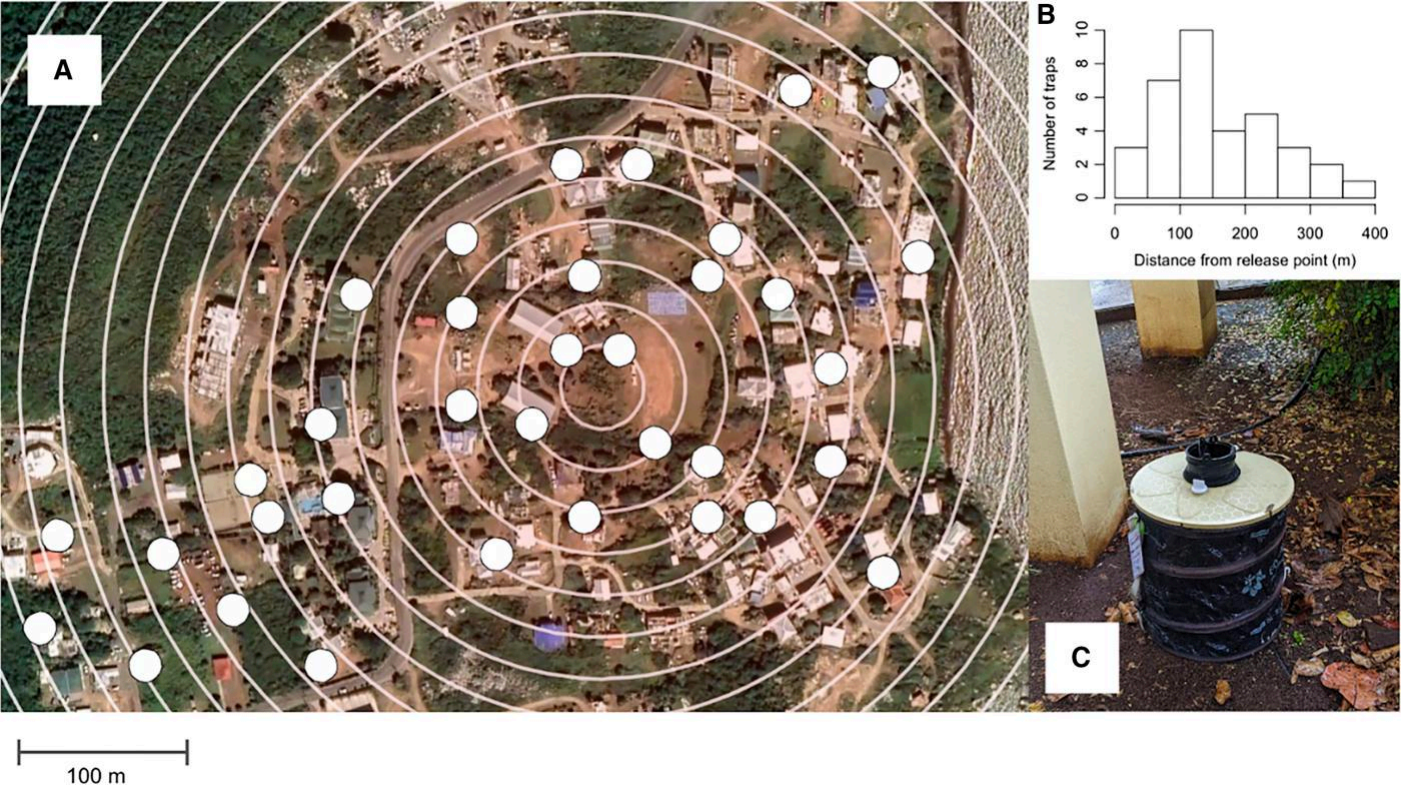
Placement and geographical distribution of the 35 Biogents Sentinel-2 (BGS2) traps in Handsome Bay around the release point. (**A**) Trapping density was targeted to be even across the site other than in vegetative areas and along the ocean border to the east. Concentric circles are drawn on the map at 25 m intervals up to 400 m from the release point. BGS2 trap locations are shown in white. (**B**) Histogram of traps by distance in meters from release point. (**C**) Photo of a BGS2 trap set with a lure at one of the designated trap locations.

MRR studies were conducted in Handsome Bay, Virgin Gorda, a neighborhood approximately 40 acres in size. The release point was located at a school in the center of the release area, 18°27′ 20.0844″ N, 64°25′ 41.3688″ W. The neighborhood is on the northern edge of Spanish Town, and consists of approximately 219 households or 118 buildings. Handsome Bay is mostly residential with a mix of multistory apartment buildings, single-family homes, and two schools. The eastern side is bordered by the ocean, with regular trade winds blowing across the neighborhood from east to west. Door-to-door community outreach, school visits, and a public informational meeting on the study were done ahead of releases and received approval from the project’s community-based steering committee. Thirty-five Biogents Sentinel-2 (BGS2) traps (Biogents AG, Germany) were placed around the release point based on a grid-mapping system to ensure approximately even trap density, up to a maximum distance of 370 m. The study site bordered the Atlantic Ocean, which limited trapping distance on the eastern edge and skewed trap placement to the west (Figure 1). Two reps of the MRR study comparing *Wb+* and/or fluorescent marked mosquitoes to WT controls were conducted in July 2022 (“MRR1-1” and “MRR1-2”) and two reps comparing *Wb+* males released directly from shipping pucks or from hand containers to WT controls were conducted in September 2022 (“MRR2-1” and “MRR2-2”). Average temperature conditions in July and September are similar with temperatures between 25 and 31°C. July and September are rainy months, considered part of hurricane season, with average rainfall 85 mm in July and 143 mm in September. In September 2022, the first rep MRR (“MRR2-1”) comparing *Wb+* males released from shipping pucks compared with containers and to WT controls was conducted during Tropical Storm Fiona with significant rainfall and cooler temperatures compared with the other weeks during which MRRs were conducted.

### *Wolbachia*-mosquito strain, production, and packing.

A strain of Virgin Gorda *Ae. aegypti* males containing *w*AlbB *Wb+* was made by backcrossing males from a WT Virgin Gorda-derived colony (“BVI-WT”) to a *Wb+*-infected DAB (Debug *Ae. aegypti* with *w*AlbB) colony backcrossed for five generations. After five generations, male progenies from the fifth generation were mated with WT females to verify cytoplasmic incompatibility and individuals from both parental strains and the newly derived backcrossed strain had DNA extracted and sequenced to compare genetic similarity. The average genetic similarity between the backcrossed strain and the WT strain was 95%. After backcrossing, the strain was maintained in laboratory conditions for five generations before MRR1-1 and MRR1-2, and seven generations before MRR2-1 and MRR2-2. Larvae were reared using automated rearing and sex sorting methods described in Crawford et al. 2020.^5^ Briefly, batches of 2,000 mosquito larvae were reared in rearing bags filled with 1.2 L of water inside a storage and retrieval robot (Verily Life Sciences, San Francisco, CA) and fed a proprietary mosquito larvae diet (1.75 mg diet per larvae). After 1 week, pupae were sieved to remove most females and transferred to an adult sex sorting machine to separate males from residual females. Adult sex sorting machines sorted 1,200 *Wb+* male mosquitoes into small containers (11.5 × 11.5 × 18.5 cm) for up to 40 hours and mosquitoes were fed 10% sucrose solution *ad libitum* during sorting. The containers with counted and sex-sorted males were then chilled to knock the mosquitoes down, and transferred into small plastic pucks (“pucks”) (70 × 70 × 23 mm) using custom Verily hardware. Pucks of live, chilled mosquitoes were placed into pressure- and temperature-controlled boxes for transit to the BVI. For releases with male mosquitoes released from hand containers, insulated boxes (40.6 × 40.6 × 50.8 cm) containing up to 33,600 mosquitoes were shipped to Virgin Gorda via air and ferry, received within 24 hours of transit, unpacked into small hand-release containers (11.5 × 11.5 × 18.5 cm) by hand, and held overnight at ambient temperature (24–29°C) with access to 10% sucrose solution until release the following day. For releases with male mosquitoes released directly from shipping pucks, the mosquitoes were packed and kept chilled and compressed identically to the mosquitoes released from hand containers during transit to Virgin Gorda, but were taken to the field to release immediately once received, without being transferred into small hand containers and without having access to an additional sugar meal. Mosquitoes released from pucks were kept inside the chilled boxes they were received in until immediately before release.

### Wild-type mosquito strain and production.

WT *Ae. aegypti* were reared in the field (Virgin Gorda EcoPark Facility) from a mixture of wild-collected eggs and eggs amplified in colony (∼F2 generation). The WT mosquitoes were reared at a similar density as the *Wb+* males reared at Verily, approximately 2 larvae per milliliter, and fed the same amount of diet per larva. Upon pupation, males were separated from females using a mechanical sieve. As males eclosed into adults, any remaining females were removed using an aspirator and groups of 1,200 WT male mosquitoes were transferred into hand containers (11.5 × 11.5 × 18.5 cm) identical to the containers used to hold the *Wb+* males. WT males were similarly fed 10% sucrose solution and kept at ambient temperatures (24–29°C) overnight before release.

### Application of fluorescent marker.

All male WT mosquitoes and a subset of shipped *Wb+* male mosquitoes were sprayed with a fluorescent marker before release based on methods developed in Faiman et al. 2021 (numbers per treatment listed in Table 1).^21^ The fluorescent dye (SmartWater CSI LLC, Great Britain) consisted of a mixture of Cartax-DP dye that fluoresces yellow-green under ultraviolet (UV) light (365 nm) and Mowilith LDM 7709, a polymer that binds the dye to the mosquito after a brief curing time. We modified the methods in Faiman et al. 2021 to use a smaller battery-pack nebulizer that was easier to use in the field and available at lower cost (ASOMI mesh nebulizer available on Amazon).^21^ The nebulizer mouthpiece was connected to a small port on the mosquito hand container and turned on to the highest setting for 30 seconds with approximately 5 mL of liquid SmartWater inside (particle size ≤4 µm, condensation rate ≥0.2 mL per minute). We used an extended spray time compared with Faiman et al. 2021 as shorter spray times resulted in harder to observe fluorescence and no detectable difference in laboratory-measured survival (Supplemental Information, Supplemental Figures 1 and 2).^21^ Each mosquito container contained approximately 1,200 adult male mosquitoes. The hand container was gently tapped during application to encourage flight. A small subset of mosquitoes was checked from sprayed containers under UV to ensure they fluoresced before release.

**Table 1:** Numbers of WT and *Wb+*-fluorescently marked, and *Wb+* unmarked *Aedes aegypti* males released and recaptured across all collection days

Treatment	Replicate	Males Shipped	Males Sprayed with Fluorescence or Held in Container	Males Released	Males Recaptured
WT fluorescent males	MRR1-1	NA	4,910	4,696	185 (3.9%)
	MRR1-2	NA	10,649	9,138	128 (1.4%)
	MRR2-1	NA	6,486	5,979	286 (4.8%)
	MRR2-2	NA	10,463	9,959	285 (2.9%)
	Total, all reps	NA	32,508	29,772	884 (3.0%)
*Wb+* fluorescent males	MRR1-1	14,400	13,277	12,901	180 (1.4%)
	MRR1-2	13,200	12,900	11,242	260 (2.3%)
	MRR2-1	10,800	10,159	9,348	413 (4.4%)
	MRR2-2	14,400	13,532	12,817	379 (3.0%)
	Total, all reps	52,800	49,868	46,308	1,232 (2.7%)
*Wb+* unmarked males	MRR1-1	13,200	12,523	12,388	309 (2.5%)*
	MRR1-2	13,200	12,828	12,477	455 (3.6%)
	MRR2-1^†^	12,000	NA	10,527	786 (7.5%)
	MRR2-2^†^	12,000	NA	10,478	573 (5.5%)
	Total, all reps	28,800	–	45,870	1,844 (4.0%)

MRR = mark-release-recapture; NA = not applicable; rep = replicate; *Wb+ = Wolbachia*; WT = wild-type. Recaptured counts are the sum of all males recaptured from all traps across all collection days.

* MRR1-1 *Wb+* unmarked samples are missing data from 87 samples from nine different traps collected day 2 because of laboratory handling error.

^†^ MRR2-1 and MRR2-2 differed from MRR1-1 and MRR1-2 in the release method of *Wb+* unmarked males. MRR2-1 and MRR2-2 released *Wb+* unmarked males directly from shipping pucks the same day as receipt, whereas MRR1-1 and MRR1-2 were released from hand containers the day after receipt.

### Laboratory validation of fluorescent marker.

Ahead of releasing marked males in the field, the fluorescent spray-marking method was evaluated in the laboratory to assess consistency in coverage of the marker on all mosquitoes in containers after spray treatment, persistence of the marker over time, impact of the marker on male longevity, and the percent of false positives and false negatives when marked males were collected in BGS2 traps with unmarked males. To assess coverage and persistence of the marker, 14 containers of ∼1,800 sorted male mosquitoes were treated with fluorescent spray as described above (“Application of fluorescent marker”). Containers were released into large BugDorm cages (45 × 45 × 45 cm) in insectary conditions (27°C, 80% relative humidity) and monitored for longevity by counting and removing dead individuals every 24 hours. Dead individuals collected on days 4, 5, and 6 after release into cages were screened for fluorescence and coverage was calculated as the percent of males with observable fluorescence under UV light out of total males screened. Persistence was evaluated by comparing the percentage of males positive for fluorescence by day collected from longevity cages. The impact of marking on male longevity was evaluated by releasing males sprayed with the fluorescent marker, or unsprayed control males, into large or medium-sized BugDorm cages (30 × 30 × 30 cm) and counting the number of dead individuals every 24 hours until death days for all individuals were recorded. We also evaluated whether marked males had the potential to transfer marking to unmarked males (“false positives”) when collected in BGS2 traps or whether marked males could potentially lose marking when stored in a BGS2 trap (“false negatives”) by releasing counted numbers of marked and unmarked males in an extra-large BugDorm cage (100 × 100 × 200 cm) with a BGS2 trap overnight. Males were then collected from the BGS2 trap, frozen for at least 2 hours, and scored for fluorescence. Numbers of fluorescent marked males versus unmarked males were compared from the collected BGS2 trap versus the count of known released males before collection.

### Mosquito release and recapture.

For each MRR rep, mosquitoes were released into the field at the release point in the center of the trapping matrix on the day after *Wb+* males were received from shipments sent from Verily Life Sciences or the day they were received for mosquitoes released directly from shipping pucks (“day 0”). As described above, *Wb+* unmarked males were transferred into release containers and held overnight at ambient temperature with 10% sucrose solution. *Wb+*-marked males were similarly transferred and held but were sprayed with fluorescent marker, described above, prior to sugar-feeding. For *Wb+*-unmarked males released directly from shipping pucks, males were kept inside the chilled boxes until they reached the release site, where the pucks were removed from packaging and opened over white trays to observe release. For all treatments, mosquitoes were given 15 minutes to disperse out of containers or shipping pucks placed over trays. Containers and pucks were gently tapped to encourage dispersal. Mosquitoes that were found dead at the bottom of release containers or that did not make it out of shipping pucks were counted and subtracted from the count of released males (Table 1). Thirty-five BGS2 traps placed in concentric circles radiating out from the release point were placed as described above (Figure 1) and turned on 24 hours after release (“day 1”) to allow the males to find natural harborage after release instead of using the traps as short-term harborage. All traps were serviced daily in the morning starting on day 2 after release (“day 2”) and up to day 6 (“day 6”) for all reps, and also on day 7 (“day 7”) for MRR2-1. Seventeen traps were run using electrical outlets and the remaining were placed on battery power (12 volts of direct current power), with fresh batteries exchanged every 3 days. All BGS2 traps were baited with a BG cartridge lure (Biogents, Germany) without carbon dioxide (CO_2_). Fan speed and trap errors were recorded during servicing to account for any malfunctioning traps. Clean catch nets were replaced in traps after collecting and the collected catch nets were placed in a cooler with ice for transport back to the laboratory. Collections were tracked to trap location and collection date using printed quick response barcode labels scanned using the AmigoCollect app (AmigoCloud, Seattle, WA). At the laboratory, collected catch nets were frozen for 2 hours to kill any live mosquitoes in the collection and then sorted using a light microscope to species and sex. Mosquitoes identified as male *Ae. aegypti* were scored as fluorescent or not using a UV flashlight (365 nm wavelength, uvBeast, Portland, OR) in a dark room. All data were recorded in custom Verily software. Samples collected from MRR1-2 were significantly impacted by ant damage as evidenced by some collections having only partially intact mosquitoes in the catch net. We recorded a collection as damaged by ants when ants were found in the collection net and more than 50% of mosquitoes in the collection were missing body parts or were only identified by their thorax. Ant damage was most prevalent in MRR1-2 (12 of 210 (6%) collections were damaged by ants in MRR1-2 compared with two in MRR1-1 (<1%), four in MRR2-1 (2%), and two in MRR2-2 (<1%). Maps of collected mosquitoes by location were made using the R “googleway” package.^27^

### *Wolbachia* detection via a loop-mediated isothermal amplification (LAMP) assay.

Collected *Ae. aegypti* males screened for fluorescence were placed in collection tubes and shipped to Verily Life Sciences for molecular detection of *Wb+*. MRR1-1 collections were shipped on ice and delays in transit resulted in many samples with observed mold on arrival. MRR1-2, MRR2-1, and MRR2-2 were placed in tubes with silica gel (Dry and Dry orange indicating gel, Brea, CA) and cotton wool to avoid microbial growth and preserve DNA without the need to ship on ice. DNA from all samples was extracted using the Extracta DNA prep kit (Quantabio, Beverly, MA). Briefly, individual mosquito samples were plated on 96-well Axygen 1.1 mL deep-well plates with sample identification (ID) traced to fluorescent screening result, trap ID, and collection date. Each well had 50 µL extraction buffer, a single steel bead (Fisher Scientific, 02-215-512, Waltham, MA), and the individual mosquito sample. Plates were sealed with silicone mats (Axygen, AXYMAT AM-2ML-RD). Samples were homogenized for 2 minutes at 900 revolutions per minute using a SPEX homogenizer and then transferred to an Olympus 96-well polymerase chain reaction (PCR) plate. PCR plates with homogenate were run on a thermocycler (Biorad T100) at 95°C for 30 minutes and then held at 22°C. Samples were then spun down. Fifty microliters of stabilization reagent were added to each well once samples reached room temperature. Plates were sealed, shaken, spun down, and then transferred to a NUNC DNA plate and stored at −20°C. Extracted DNA was used to test for the presence of *Wb+* using a LAMP assay, which was designed to detect the *Wb+*-specific 16S ribosomal RNA gene. Primer sequences and the LAMP protocol used were the same as in Crawford et al. 2020.^5^ For each 25 µL reaction, 1 µL of sample DNA was added to 12.5 µL WarmStart Colorimetric LAMP 2X Master Mix (DNA and RNA) (New England BioLabs, M1800, Ipswich, MA), 8.32 µL water, 2.5 µL of 10X primer mix (concentration of F3 and B3 was 2 µM each in 10X stock, forward inner primer and backward inner primer were each 16 µM in 10X stock), along with 0.5 µL Antarctic Thermolabile UDG (New England BioLabs, M0372) and 0.18 uL deoxyuridine triphosphate (Thermo Fisher, FERR0133) to prevent carryover contamination. Reactions were prepared on ice in 96-well PCR plates (Olympus Plastics, Genesee Scientific, 24-300, Morrisville, NC), then incubated at 68°C for 1 hour 30–45 minutes, 85°C for 5 minutes, and cooled to 4°C on thermal cyclers (Bio-Rad T100, Hercules, CA). At least one non-template control, along with a positive and negative control mosquito, were loaded on every plate before the DNA extraction phase. Samples were visually scored for color change to distinguish positive versus negative samples. Positive samples appeared yellow. Negative samples appeared pink.

### Mating competitiveness of packed and shipped *Wolbachia* males compared with wild type.

To evaluate the mating competitiveness of packed and shipped *Wb+* males compared with WT, mating assays were conducted to estimate Fried’s index, *c*, a mating competitiveness coefficient that describes the odds that a female will mate with an incompatible *Wb+* male over a WT male.^28^ A value of *c =* 1 denotes equal competitiveness, whereas a value of *c <*1 denotes lower competitiveness of *Wb+* males relative to WT, and a value of *c >*1 denotes higher competitiveness of *Wb+* males to WT. Fried’s index is estimated based on the average hatch rate of eggs from individual females by using the formula: *c* = (*w/second*) × [(*H_w_* − *H_c_*)/(*H_w_* − *H_s_*)] where *w* denotes the number of WT males, *s* denotes the number of *Wb+* males, and H denotes the hatch rate of either females mated to males in competition cages with both *Wb+* males and WT males (*H_c_*), in cages with only *Wb+* males mated to WT females (*H_s_*) (incompatible control), or in cages with only WT males (*H_w_*) (compatible control). To estimate *H_c_*, three reps with 10 packed and shipped *Wb+* males in competition with 10 WT males were set up in large-sized BugDorm cages (32.5 × 32.5 × 32.5 cm) and allowed to mate for 24 hours with 10–20 WT females. Packed and shipped *Wb+* males were collected following Verily standard production release protocols, described above. WT mosquitoes were reared on-site and were sex-separated microscopically and then separately held in cages with access to 10% sucrose ad libitum. After release, males had access to reverse osmosis (RO) water only. *Wb+* males were tested independently in three biological reps and three technical mating cages per biological rep. For each cage, 10 *Wb+* males were combined with 10 age-matched WT and allowed to acclimatize for 1 hour. Ten (10) WT females were then added to the cage and males and females allowed to mate for a 24-hour period. The females were then removed from the cage and provided with a blood meal. Females were allowed to oviposit individually 48 hours after blood feeding by placing into iso-cups with RO water and seed germination paper. Two (2) days after oviposition the germination papers containing eggs were stored in plastic containers with the lids ajar and allowed to dry for a minimum of 3 days, after which the eggs were encouraged to hatch by flooding papers with hatching broth (per 1L RO water: 0.071 g yeast and 0.357 g nutrient broth). Flooded egg papers were maintained under insectary conditions (28°C, 80% relative humidity, 12:12 light cycle) for 3 days and then eggs were assessed microscopically and unhatched/hatched eggs counted. One compatible and one incompatible cage per biological rep were run with a total of 20 males and 10 females and eggs collected and assessed for hatching as described above.

## STATISTICAL ANALYSES

Survival data were analyzed using Cox proportional hazard models in the R “survival” package.^29^ Recapture rates were calculated as the proportion of total recaptured males out of the total number of released males for each treatment and rep. Statistical significance (*P*-value <0.05) was evaluated using the Kruskal-Wallis test to compare recapture rates between treatments and reps. Mean distance traveled (MDT) was calculated as in previous studies using a correction factor to account for uneven distribution of traps across the release area.^10^^,^^30^ Differences between dispersal distances among reps and treatments were compared using generalized linear model fits on individual mosquito dispersal distances including either no fixed effects, or rep, treatment, or an interaction between rep and treatment as fixed effects (‘glm’ function in R using family = Gamma [link=‘log’] as data were not normally distributed). Model fits were compared based on Akaike Information Criterion (AIC), with the best fit model selected based on its lowest AIC value; deltaAIC >2 were considered significantly different. PDS and estimated recapture rates adjusted for mortality were estimated using the nonlinear least squares (nls) regression described in Buonaccorsi et al. 2003,^31^ modified to constrain recapture rates below 10%. Trap efficiency above 10% for BGS2 traps without CO_2_ is not considered realistic; field estimates of trap efficiency with CO_2_ have been observed around 9% and BGS2 traps without CO_2_ are expected to have lower efficiency.^32^^,^^33^ Parameter constraints were applied using the nls function in R, specifying the ‘port’ method and constraining recapture estimates to be between 0 and 10% (R version 4.2.3, The R Foundation for Statistical Computing, 2023). The port method is from the R port library and uses an adaptive nls algorithm, ‘NL2SOL’.^34^ Parameter estimates for PDS were not constrained, i.e., allowed to vary between 0 and 100%. CIs were calculated using bootstrap resampling (*B =* 10,000), as described in Buonaccorsi et al. 2003.^31^ The average life expectancy was defined as 1/-ln(PDS).^35^ Statistical comparisons of PDS across rep and treatments were done by rejecting the null hypothesis that PDS was equivalent for treatments and/or reps where bootstrap 95% CIs did not overlap (*P*-value <0.05).^31^ Estimates of population size were calculated using the Lincoln-Peterson Index.^36^^,^^37^

## RESULTS

### Laboratory validation of fluorescent marker.

Containers of mosquitoes marked with fluorescent marker showed nearly complete coverage and persistence of fluorescence on mosquitoes out to 7 days post-marking (98–100%, Supplemental Table 1). Mosquitoes marked or unmarked with fluorescent marker solution showed no difference in laboratory-conducted survival assays (hazard ratio from Cox proportional hazard models was not significantly greater than 0, 1.09, 0.96–1.24, 95% CI for marked mosquitoes compared with unmarked controls, Supplemental Figures 1 and 2). Number of mosquitoes marked or unmarked with fluorescent solution and released into cages with BGS2 traps showed no evidence that fluorescence was lost or transferred from storing unmarked and marked mosquitoes together (Supplemental Table 2).

### Release counts and recapture rates.

**Figure 2.**
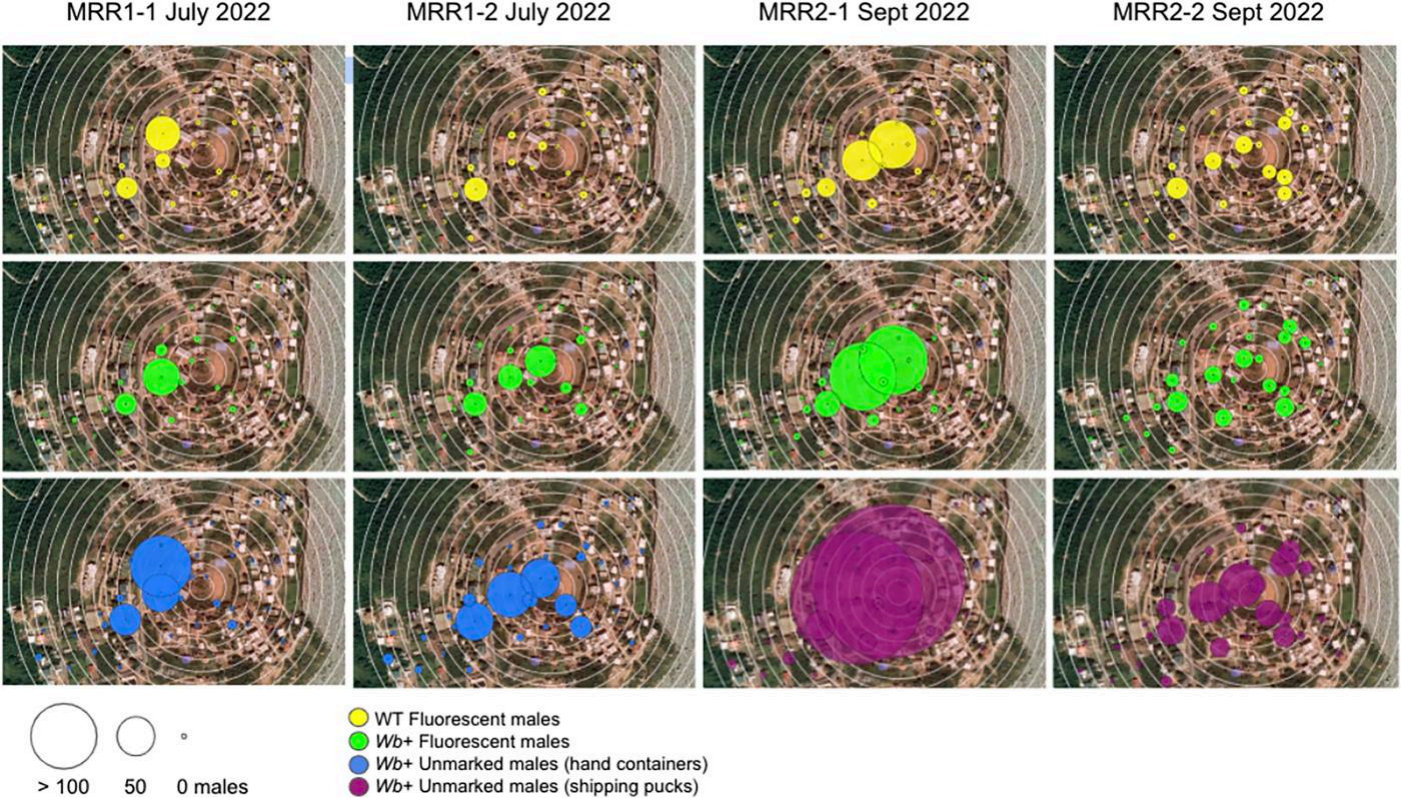
Recaptured male mosquitoes by treatment and trap location. Thirty-five Biogents Sentinel-2 (BGS2) traps were placed within a 400 m radius of the release location (center of map). Concentric circles drawn around the release location are drawn at 25 m intervals. Total males recaptured in each location after 6 or 7 days of sampling after release were similar within replicates (reps) for fluorescently marked males with or without *Wolbachia* (*Wb+*) but consistently higher for unmarked males. The sum of total recaptures by rep are highlighted in yellow for wild-type (WT) fluorescent males released from hand containers, green for *Wb+* fluorescent males released from hand-containers, blue for *Wb+* unmarked males released from hand containers, and magenta for *Wb+* unmarked males released directly from shipped pucks.

**Figure 3.**
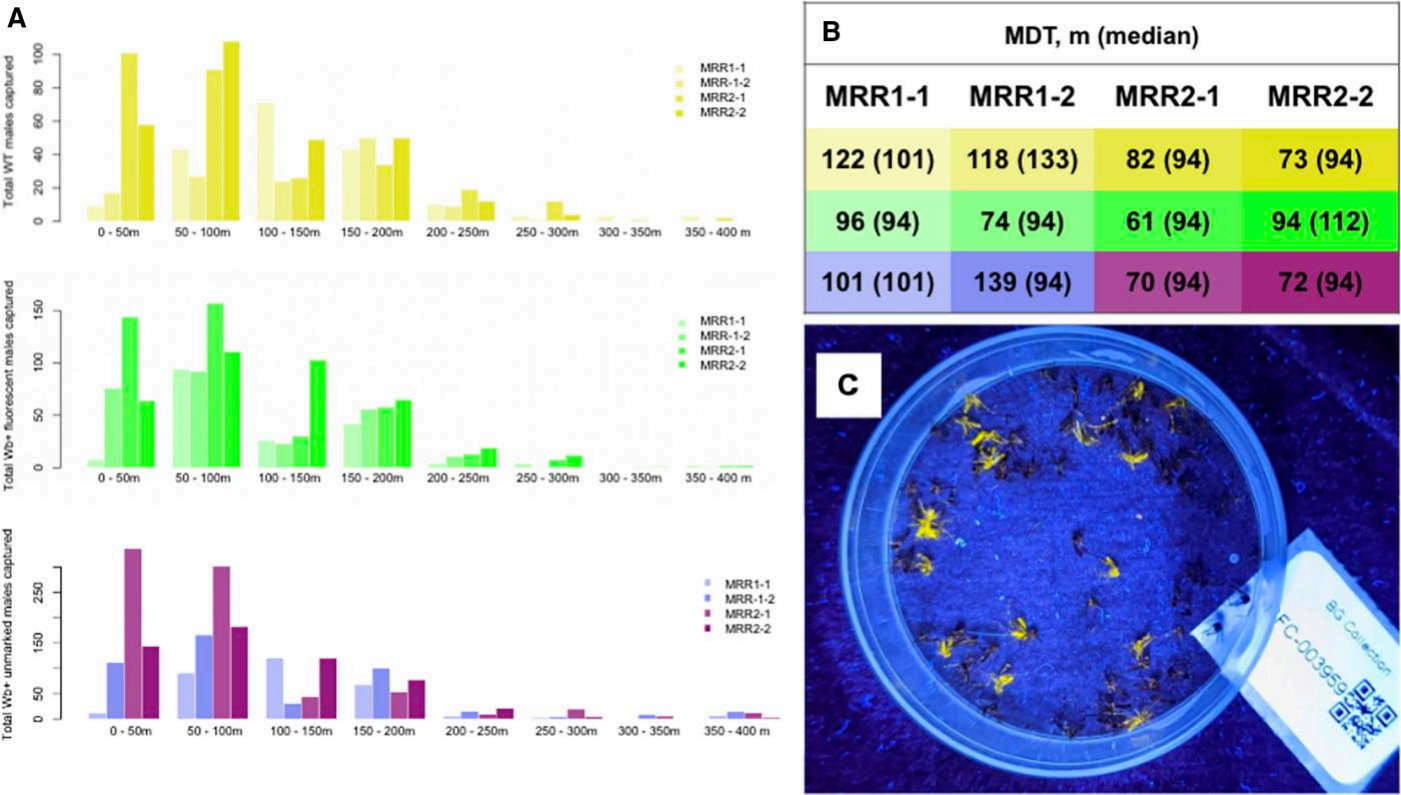
Distribution of male mosquitoes caught by trap distance in each treatment and replicate (rep). (**A**) Histogram of total numbers of mosquitoes caught by distance away from the release point with wild-type (WT) fluorescent males shown in yellow, *Wolbachia* (*Wb+*) fluorescent males in green and *Wb+* unmarked males shown in blue for males released from containers and magenta for males released directly from shipping pucks. Different replicates are shown in different shades. (**B**) Adjusted mean distance traveled (MD) in meters by rep and treatment corresponding to the same color legend in panel A. Median values are in parentheses. (**C**) Image of fluorescent-marked males and unmarked males under ultraviolet (UV) light.

In total, 29,772 fluorescent WT males, 46,308 fluorescent *Wb+* males, and 45,870 unmarked *Wb+* males were released across four reps in July and September 2022. Released mosquitoes were approximately the same age (3 days after eclosion for *Wb+* unmarked mosquitoes released directly from shipping pucks and 4 days after eclosion for all other treatments). We collected 9,239 *Ae. aegypti* males, 5,176 *Ae. aegypti* females, 10,501 *Culex* males, and 11,197 *Culex* females in total from all collections (distribution of recaptured males by location shown in Figure 2; distribution by median distance travelled shown in Figure 3A and summarized in Figure 3B; appearance of fluorescent males under UV shown in Figure 3C). Of the 9,239 total *Ae. aegypti* males collected, 2,113 scored positive for fluorescence, 3,358 scored positive for *Wb+*, and 1,232 scored positive for both fluorescence and *Wb+* (Table 1). Recapture rates were between 1.4–7.5% and averaged 3.2% across reps and treatments. These values were not adjusted for mortality (see “recapture rates adjusted for PDS” below). Recapture rates were not statistically different between fluorescent and not fluorescent males (Kruskal-Wallis χ^2^ = 1.85, degrees of freedom [df] = 1, *P*-value = 0.17), reps (Kruskal-Wallis χ^2^ = 6.14, df = 3, *P*-value = 0.11), *Wb+* and WT males (Kruskal-Wallis χ^2^ = 0.065, df = 1, *P*-value = 0.80), nor treatment comparisons of *Wb+* fluorescent males, *P*-value = 0.35).

### Dispersal distance.

The majority of recaptured males were captured between 0–200 m (Figures 2 and 3). MDT ranged from 61 to 139 m depending on treatment and rep (Figures 2 and 3). MDT ranged from 94 to 133 m (Figure 3B). The best fit ‘glm’ model fit of dispersal distances accounted for an interaction between rep and treatment (dispersal distance∼treatment*rep). To evaluate the effect of treatment within each rep, we fit glm models (dispersal distance∼treatment) for each rep. There was no significant effect of treatment in MRR1-1 and MRR2-1; all treatments had equal dispersal away from the release point. In MRR1-1, this was marginally skewed toward WT males dispersing further than *Wb+* males (*P* = 0.09), and in MRR2-1, there was a marginal effect of unmarked *Wb+* males dispersing shorter distances (*P* = 0.07). In MRR1-2, fluorescent *Wb+* males dispersed shorter distances than WT males and unmarked *Wb+* males (*P* <0.005). In MRR2-2, fluorescent *Wb+* males dispersed further than WT (*P* <0.05) and unmarked *Wb+* males released directly from pucks (*P* <0.005).

### Probability of daily survival (PDS) and recapture rates adjusted for PDS.

**Figure 4.**
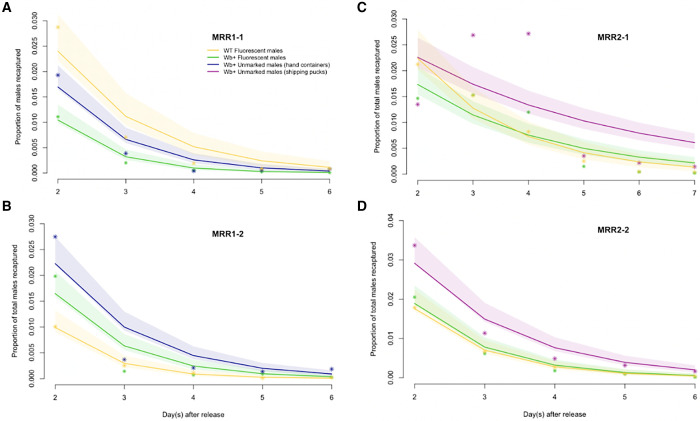
Actual and estimated recaptured males by day after release. Nonlinear least squares (NLS) model fits (lines) and CIs (shaded area) for estimated proportion of recaptured males, as well as empirical data proportions (points) by treatment and rep (replicate) are plotted by day post-release. Wild-type (WT) fluorescent males are shown in yellow, *Wolbachia* (*Wb+*) fluorescent males in green, and *Wb+* unmarked in light blue (container release, mark-release-recapture [MRR]1) or magenta (puck release, MRR2). (**A** and **B**) Correspond to reps MRR1-1 and MRR1-2. (**C** and** D**) Correspond to reps MRR2-1 and MRR2-2.

Recapture rates adjusted for differential mortality were similar among treatments and reps (Table 2). Only *Wb+* males in MRR2-1 had significantly lower recapture rates than in other treatments and reps. PDS estimates from the nls model fits were significantly higher for *Wb+* unmarked males compared with fluorescent *Wb+* males in all reps (Table 2). In MRR1-1, fluorescent Wb+ males had lower PDS than fluorescent WT males, but the opposite result was observed in MRR1-2, and no effect of WT versus *Wb+* was detectable in MRR2-1 or MRR2-2 (Table 2). Model fits of the estimated males recaptured by day with 95% CIs are compared with the raw data in Figure 4.

**Table 2:** NLS model estimates of PDS and adjusted recapture rates

Replicate	Treatment	Recapture Rate Adjusted for PDS, nls	PDS Estimate, nls	Average Life Expectancy, Days
MRR1-1	WT fluorescent males	0.10 (0.07–0.10)	0.52 (0.52–0.58)	1.5 (1.5–1.8)
	*Wb+* fluorescent males	0.10 (0.07–0.10)	0.34 (0.34–0.41)	0.9 (0.9–1.1)
	*Wb+* unmarked males*	0.10 (0.08–0.10)	0.43 (0.44–0.49)*	1.2 (1.2–1.4)
MRR1-2	WT fluorescent males	0.10 (0.06–0.10)	0.33 (0.33–0.41)	0.9 (0.9–1.1)
	*Wb+* fluorescent males	0.10 (0.08–0.10)	0.43 (0.43–0.48)	1.2 (1.2–1.4)
	*Wb+* unmarked males	0.10 (0.08–0.10)	0.50 (0.50–0.54)	1.4 (1.4–1.6)
MRR2-1	WT fluorescent males	0.06 (0.05–0.09)	0.64 (0.56–0.66)	2.2 (1.7–2.4)
	*Wb+* fluorescent males	0.04 (0.03–0.05)	0.68 (0.64–0.73)	2.6 (2.2–3.2)
	*Wb+* unmarked males^†^	0.04 (0.03–0.05)	0.80 (0.77–0.83)^†^	4.5 (3.8–5.4)
MRR2-2	WT fluorescent males	0.10 (0.08–0.10)	0.44 (0.44–0.50)	1.2 (1.2–1.4)
	*Wb+* fluorescent males	0.10 (0.08–0.10)	0.46 (0.46–0.51)	1.3 (1.3–1.5)
	*Wb+* unmarked males^†^	0.10 (0.09–0.10)	0.58 (0.58–0.61)^†^	1.8 (1.8–2.0)

MRR = mark-release-recapture; nls = nonlinear least squares; PDS = probability of daily survival; *Wb+ = Wolbachia*; WT = wild-type. Recapture rates estimated from the nls regression were constrained to be at or below 10% with 95% CIs shown in parentheses. Corresponding PDS estimates with 95% CIs are listed by replicate and treatment. Average life expectancy in days was calculated based on the PDS estimates and CI intervals given.

* MRR1-1 *Wb+* unmarked samples are missing data from 87 samples from nine different traps collected day 2 because of laboratory handling error.

^†^ MRR2-1 and MRR2-2 differed from MRR1-1 and MRR1-2 in the release method of *Wb+* unmarked males. MRR2-1 and MRR2-2 released *Wb+* unmarked males directly from shipping pucks the same day as receipt, whereas MRR1-1 and MRR1-2 were released from hand containers the day after receipt.

### Mating competitiveness of packed and shipped Wolbachia males compared with wild-type.

*Wb+* males released from hand containers were directly compared with WT males in their ability to mate with WT females in cage assays. Fried’s index was calculated by averaging the hatch rates from each technical rep (nine technical reps) to ensure that each rep gets the same weight, independent of the number of females that laid eggs, and using the formula from Fried, 1971.^28^ A total of 44, 18, and 13 females in competitive, compatible, and incompatible cages, respectively, laid eggs and were used in the analysis. *H_w_*, *H_s_*, *H_c_* were calculated experimentally to estimate Fried’s index of mating competitiveness. Females from the WT mating cages collected a total of 830 eggs, from which 558 hatched, an average hatch rate of 54% or *H_w_* = 0.54 (282 of 389 or 50% hatched from Rep 1, 101 of 186, or 43% hatched from Rep 2, and 175 of 255 or 70% hatched from Rep 3). Females mated with incompatible *Wb+* males laid a total of 811 eggs, from which 0 eggs hatched, an average hatch rate of 0% or *H_s_* = 0 from all reps (zero of 238 or 0% hatched from rep 1, zero of 233 or 0% hatched in rep 2, and zero of 340 or 0% hatched from rep 3). Females mated in competition cages with *Wb+* and WT males had hatch rates (*H_C_*) of 11% in rep 1 (96 of 647 eggs hatched), 38% in rep 2 (220 of 582 eggs hatched), and 32% in rep 3 (380 of 1,243 eggs hatched), which translated to an aggregate Fried’s across all reps of *c =* 0.98.

#### Population size.

The number of males released and recaptured was used to estimate the size of the WT population, using the Lincoln-Peterson Index as described in Mercer et al. 2012.^38^ Based on our recapture rates, we estimated that the wild-population size of *Ae. aegypti* males in Handsome Bay, Virgin Gorda, was between 1,991 and 3,119 wild males per hectare while conducting the MRR studies using the Lincoln-Peterson Index (2,900 males per hectare for MRR1-1, 2,426 males per hectare for MRR1-2, 1,991 males per hectare in MRR2-1, and 3,119 males per hectare in MRR2-2).

## DISCUSSION

Recapture rates of released *Ae. aegypti* males averaged 3.2% using BGS2 traps with lures and without CO_2_. Recapture rates in this study were similar or higher than observed in previous studies for male *Ae. aegypti* MRRs using BGS2 traps without CO_2_ (0.04–0.07% in Marina et al. 2022, 4.4% in Neira et al. 2014, 1.6–3.2% in Maciel-De-Freitas 2007, not reported in Spinner et al. 2022) and similar to those observed in *Wb+* male MRRs recaptured with CO_2_ (3–6% in Mains et al. 2019) despite not using CO_2_ in this study.^3^^,^^11^^,^^12^^,^^35^^,^^39^^,^^40^ Recapture rates adjusted for mortality were between 4% and 10%, but we artificially constrained nls model estimates for the recapture rate to be below 10% as previous work on trap efficiency has suggested that the maximum efficiency is around 9%, with efficiency defined as the percent of approaches that results in a capture.^32^^,^^33^ Recapture rates and rates adjusted for mortality did not differ significantly between *Wb+* male mosquitoes and WT in most reps, nor were recapture rates impacted by fluorescent marking (Tables 1 and 2), suggesting that the method of fluorescence marking we used did not impact recapture or bias comparisons between WT-marked males and unmarked *Wb+* males.

Dispersal distances of WT males compared with *Wb+* males with or without fluorescence did not significantly differ in our study in reps MRR1-1 and MRR2-1, despite the ongoing tropical storm in MRR2-1. In MRR2-2, unmarked *Wb+* males showed reduced dispersal compared with fluorescent *Wb+* males, and this trend was also marginally significant in MRR2-1 (Figure 3). Unmarked *Wb+* males in MRR2-1 and MRR2-2 were released from shipping pucks immediately upon receipt in the BVI after shipping for 24 hours from the United States. This meant that *Wb+* unmarked males in MRR2-1 and MRR2-2 were released while cool from being packed and chilled without time to recover before release, and all mosquitoes in these reps were released later in the day than in MRR1-1 and MRR1-2. Packed and chilled mosquitoes take some amount of time to recover after packing and shipping, which could have reduced their ability to disperse, especially in the first day after release. Unmarked *Wb+* males also were not given additional sugar on receipt as they were released directly from shipping pucks in MRR2-1 and MRR2-2, whereas the fluorescent marked *Wb+* males were held overnight with sugar before release. The additional sugar meal given to the fluorescent-marked mosquitoes released from hand containers in MRR2-1 and MRR2-2 could have benefited their dispersal compared with unmarked males. MRR1-2 showed reduced dispersal of fluorescent *Wb+* males compared with WT males and unmarked *Wb+* males, which might be expected if there was a cost of *Wb+* and fluorescent marking on dispersal distance, but this was not seen in any other rep. MRR1-2 had the lowest recapture rates of all reps (2.4% average recapture rate compared with 2.6–5.6% in other reps) and many collections were damaged by ants, suggesting this finding could have been caused by spurious results from lower sample sizes and some missing data because of ant damage (12 of 210 [6%] collections were damaged by ants in MRR1-2 compared with two in MRR1-1 [<1%], four in MRR2-1 (2%), and two in MRR2-2 [<1%]). Mean dispersal distances ranged from 61 to 139 m and were similar to or higher than values estimated for male *Ae. aegypti* in other locations (55 m in Spinner et al. 2022, 56 m in Muir and Kay 1998, and 44–575 m in Mains et al. 2019).^3^^,^^12^^,^^41^ Maps of where our recaptured males were recovered showed a westward bias in where males in our study were captured, suggesting that wind, which blows east to west in our study site, may have influenced dispersal (Figure 2). Our dispersal estimates of >50 m for most mosquitoes suggests that mosquito release points during an IIT program can safely be spaced as far as 100 m apart with mosquitoes dispersing between points to provide even coverage over an IIT treatment area.

Survival (PDS) estimates were not significantly different between WT males and *Wb+* males in most reps (PDS not significantly different in all reps except for MRR1-1), suggesting that *Wb+* males packed and shipped over 3,000 miles can survive in a foreign environment similar to locally reared, WT males, an important indicator for success of an IIT program. PDS estimates were higher for *Wb+* unmarked males released directly from shipping pucks in MRR2 and were consistently lower for fluorescently marked *Wb+* males compared with unmarked *Wb+* males in MRR1 and MRR2. We present a comparison of the model fit and the raw data for all reps in Figure 4. The model fits to the raw data were not equally good for all reps, and were particularly poor for MRR2-1, where we saw an increase in males recaptured on day 3 post-release instead of an expected decrease in males recaptured over time (Figure 4). Fitting models to field data presents difficulty in that unexpected variables (e.g., weather, ant damage) can change the expected distribution of the data and make for a poor model fit. Despite these challenges, the nls model used presented a better fit to the raw data than alternative linear or linear-corrected models, as discussed in Buonaccorsi et al. 2003.^31^ The results from the PDS estimates of the nls models suggest that *Wb+* males are competitive in the metric measured compared with WT, and that fluorescent-marking males can inflict survival costs (Table 2). We consistently saw lower PDS estimates for marked males compared with unmarked males in every rep, suggesting that the way we marked males reduced their survival in this study (Table 2). The lack of consistent differences in PDS between marked *Wb+* males and WT males suggested that packed and shipped *Wb+* males can survive similarly to locally reared WT, which is encouraging evidence for their ability to survive during an IIT release program. Finally, PDS estimates for unmarked *Wb+* males compared between MRR1 to MRR2 suggest that releasing mosquitoes directly from pucks was similar or better than releasing from containers (PDS estimates were similar or higher for *Wb+* unmarked males in MRR2-1 and MRR2-2 compared with MRR1-1 and MRR1-2). Releasing *Wb+* males directly from shipping pucks instead of releasing males into containers, holding with access to a sugar meal, and then releasing the following day, saves time, money, and reduces handling of male mosquitoes. In addition, the released males were ∼1 day younger on release. Mortality during the unpacking procedure into hand containers and the risk of mosquitoes becoming stuck in the hand containers are also eliminated (Table 1). The lack of an additional sugar meal did not seem to negatively impact PDS for these males. PDS estimates were higher in MRR2-1 than in other reps (Table 2). MRR2-1 took place during Tropical Storm Fiona in September 2022 and had cooler, rainier, and windier weather than in other reps. The cool and stormy weather could have contributed to higher survival estimates, either from direct temperature-dependent influences on survival or from reduced flight and less associated mortality risk during the storm. The lack of any evidence for negative impacts in PDS, dispersal distance, or raw recapture rates for MRR2-1 during Tropical Storm Fiona, compared with other reps not conducted during tropical storms, suggests that male mosquitoes can persist through extreme weather events (e.g., rain, wind). Males likely take harborage during stormy weather (which we observed by reduced recapture on day 2 post-release, Figure 4), but were then able to resume normal patterns of dispersal and survival (observed by an increase in males recaptured on day 3 post-release, Figure 4).

Overall, PDS estimates and average life expectancy were similar to estimates from other MRR studies of male *Ae. aegypti* (0.9–4.5 days in our studies compared with 1.3 days in Spinner et al. 2022, 0.5–2.3 days in Neira et al. 2014, and 5.8 days in Trpis and Hausermann 1986) and suggests that most released males during a *Wb+* male release program for IIT will not survive more than a few days after release.^12^^,^^40^^,^^42^ These estimates imply that more frequent releases per week are necessary to maintain consistent overflooding ratios between released males and WT males in a release program, i.e., two to three releases per week instead of one release per week. The nls model used by Buonaccorsi et al. assumes that the system is closed and males only escape through capture or death.^31^ Migration outside of the trapping area could be a method of escape not accounted for in the model, and might bias survival lower for mosquitoes in our study with longer dispersal distances that are not accounted for in the model.

The number of males released and recaptured in MRR studies were also used to provide estimates on the size of the WT population, useful for understanding numbers needed to achieve sufficient overflooding ratios in an IIT release program. Based on our recapture rates, we estimated that the wild-population size of *Ae. aegypti* males in Handsome Bay, Virgin Gorda, was between 1,991 and 3,119 wild males per hectare while conducting the MRR studies using the Lincoln-Peterson Index (2,900 males per hectare for MRR1-1, 2,426 males per hectare for MRR1-2, 1,991 males per hectare in MRR2-1, and 3,119 males per hectare in MRR2-2). These values are several-fold higher than estimates from other studies (12–81 males per hectare in Neira et al. 2014, and 37–107 males per hectare in Trpis and Hausermann 1986).^40^^,^^42^ These estimates depend on a range of assumptions and may be overly simplistic, especially in study sites like the one presented here where the population is not closed and could be sensitive to immigration. Regardless, the high values highlight the need for additional methods of source reduction and mosquito control efforts ahead of *Wb+* males releases for IIT to sufficiently control a large wild population and achieve a target of ∼10:1 released males to WT. They also provide a rough approximation for how mosquito population size relates to the number of mosquitoes caught per trap night in a BGS2 trap. Using complimentary control methods such as trapping adults out, wide-area larvicide spraying, augmenting *Wb+* males with insecticides, and/or traditional larval source management are useful to consider to enhance the impact of released *Wb+* males by reducing competition from WT males and increasing the overflooding ratio without changing release rates.^43^^–^^48^

Our results suggest that packed and shipped *Wb+* male mosquitoes did not differ significantly from WT males marked with the same fluorescent marker (no significant differences in recapture rates, survival, or dispersal between fluorescent *Wb+* males compared with fluorescent WT males) and suggest that packing and shipping *Wb+* males from the West Coast of the United States to a field release site in the BVI can be a viable solution for delivering healthy, competitive males for an IIT program. Males used in this study were packed, shipped, and received within a 24-hour transit time. We did not evaluate longer periods of transit time, which may negatively impact performance of released males and which could occur with shipment delays or when shipping to release locations further away from a central production facility.

In MRR1 which only released males from hand containers, fluorescent-marked *Wb+* mosquitoes behaved similarly to unmarked *Wb+* mosquitoes in dispersal distance. Recapture rates were also similar in MRR1 and MRR2 between *Wb+* males with and without fluorescence. *Wb+* marked males dispersed shorter distances compared with *Wb+* unmarked males in MRR2, but this was not consistent in MRR1, where there was no significant difference between treatments. These results suggest that fluorescent-marked males suffer limited if any impairment in flight distance from marking using the methods from Faiman et al. 2021.^21^ However, PDS estimates were consistently lower in every rep for *Wb+*-marked males compared with unmarked *Wb+* males, suggesting that despite the laboratory-based data showing no impairment in survival (Supplemental Information and in Faiman et al. 2021), there were survival costs to marking for mosquitoes released in the field.^21^ It’s possible that modifications to the method presented in Faiman et al. 2021 influenced our results and further adjustment of these methods could result in less impact on survival in the field in future studies (i.e., reducing spray time or volume to reduce amount of fluorescence on mosquitoes). We chose conservative adjustments to the original protocol to ensure that there was sufficient fluorescence to avoid false negatives if fluorescence were to wear off over time in field conditions or in the BGS2 traps used for collections.

Results from MRR studies alone are not sufficient for predicting competitiveness of released males for an IIT program. Mating competitiveness is also an important indicator of success and is a component of fitness that could be impacted by packing and shipping males to remote locations. We additionally ran laboratory studies comparing mating competitiveness of packed and shipped *Wb+* males to WT controls to evaluate Fried’s index, an estimate of mating competitiveness. Across three reps of competition cages, we saw an average Fried’s index of *c =* 0.98, or nearly equivalent mating competitiveness between WT and packed and shipped *Wb+* males. These data provide additional evidence that packing and shipping has minimal impact on fitness of males intended for release in a *Wb+* IIT program and should be equally likely to mate with WT females as WT males.

## CONCLUSION

Overall, our results provide encouraging evidence that packed and shipped *Wb+* males can be competitive with WT males for an IIT program in Virgin Gorda or in other remote locations by shipping mosquitoes from a centralized production facility. Given the high population size estimates of the wild population from the Lincoln-Peterson Index in the area we conducted the MRR studies, source reduction work ahead of releases would be useful for improving overflooding ratios of released males. Fluorescent-marked mosquitoes using the method from Faiman et al. provides a reliable and easy marker to use in remote field sites for future MRR studies with BGS2 traps, but comes with the cost of reduced probability of daily survival in the field. Future studies combining the fluorescent marker with DNA barcodes could increase the number of different treatments compared in a single study and allow for further investigation of other release methods that may improve survival and dispersal of male *Wb+* mosquitoes.

Finally, our work contributes evidence to successful packing and shipping of male *Wb+* mosquitoes to remote release locations without negative impacts on mating competitiveness, survival, or dispersal of shipped males. MRR studies have not previously compared packed and shipped males from an international production facility to WT males to evaluate the impact of long-distance transport on survival and dispersal. Previous laboratory studies have shown that some methods of packing and handling can be damaging to male survival and mating competitiveness, but these impacts are minimized when transit time is less than 24 hours and depend on exact conditions of compaction and temperature.^49^^–^^51^ Our results confirm with field data that survival, recapture rates, and dispersal of packed and shipped *Wb+* males for an IIT program can be competitive with WT males when transit conditions are temperature-controlled and transit time is <24 hours. We also showed that mating competitiveness estimated using Fried’s index is comparable for packed and shipped males compared with controls. Methods for effectively packing and shipping live mosquitoes for IIT or sterile insect releases, and eggs for replacement programs, are rapidly evolving.^49^^–^^53^ Our results suggest that future programs can rely on centralized production with specialized packing and shipping systems to scale IIT programs quickly and allow for implementation in remote areas without the need for local production. Packing and shipping offers flexibility on release site locations, reduces local costs and labor in release sites, and saves cost of local production, factors which can enhance the scale and availability of IIT programs in areas with otherwise limited options for effective control of *Ae. aegypti*.

## Supplemental Materials

10.4269/ajtmh.24-0262 Supplemental Materials
