# Supplementary material for: Mark-Release-Recapture of Packed and Shipped Aedes aegypti with Wolbachia: Implications for Conducting Remote Incompatible Insect Technique Programs
Source: Am J Trop Med Hyg. 2025 Mar 18;112(6):1313–24. doi: 10.4269/ajtmh.24-0262 (PMC12139547; doi:10.4269/ajtmh.24-0262)
Supplement: Supplemental Materials [file tpmd240262.SD1.pdf]

Figure S1. Longevity of fluorescent-sprayed male mosquitoes by application time. Mosquitoes were sprayed with fluorescent marking solution in small plastic containers for variable amounts of time with no detectable cost to mosquito longevity based on application time from 0 seconds to 60 seconds. Survival plots were made using the R “survminer” package.[1]

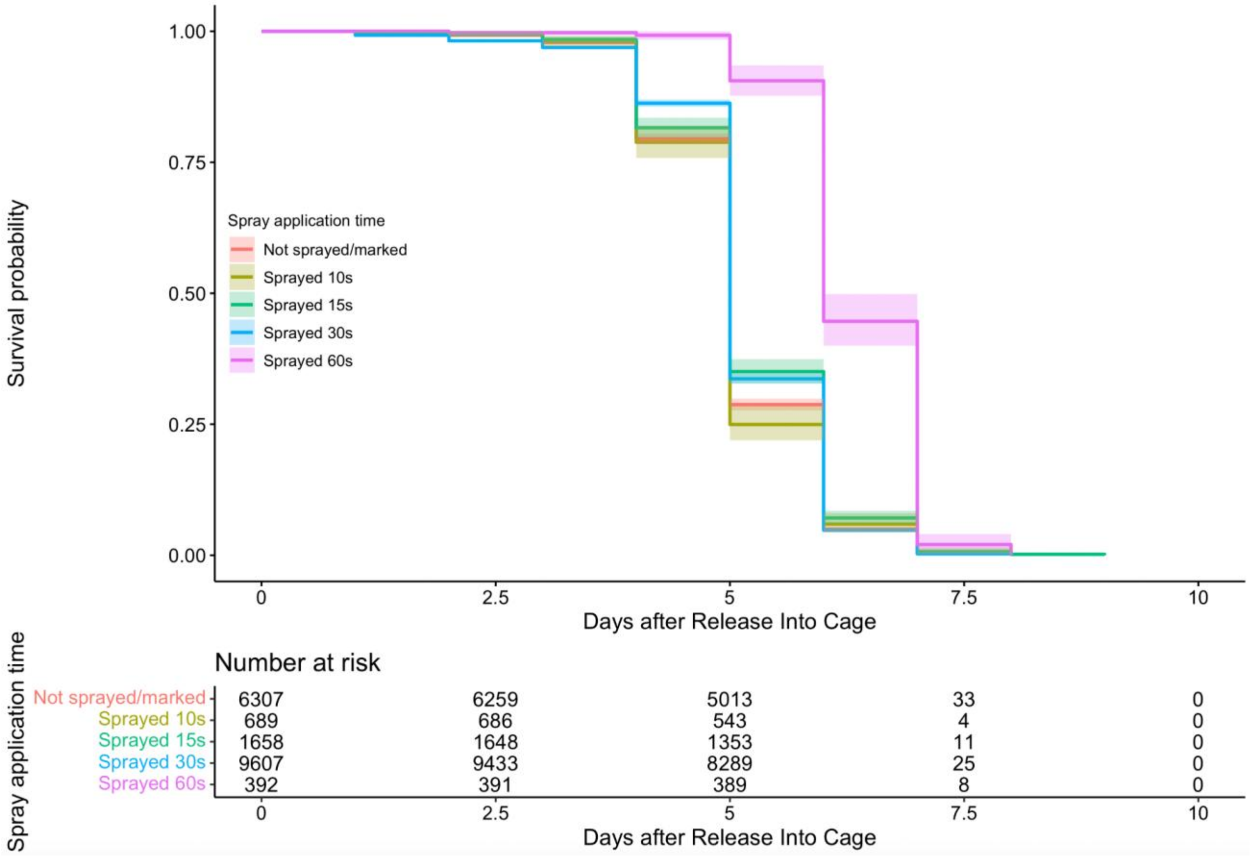

Figure S2. Longevity of fluorescent-sprayed male mosquitoes, all replicates aggregated. Marked and unmarked males showed no difference in survival in laboratory conducted survival assays. Survival plots were made using the R “survminer” package.[1]

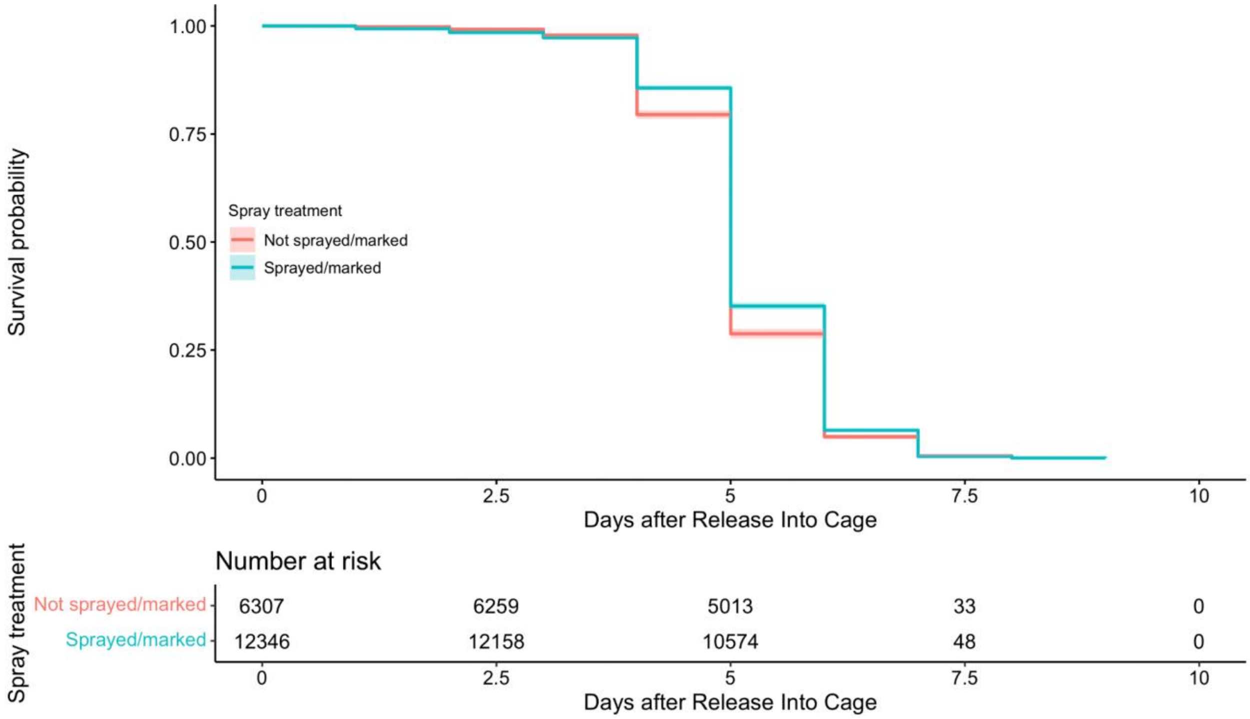

Table S1. Percent of mosquitoes covered by fluorescent spray by day after spraying. The majority (>98%) of screened mosquitoes had fluorescence after spraying with fluorescent marking solution.

Rep	# containers sprayed with subset scored	% fluorescent, day 4	% fluorescent, day 5	% fluorescent, day 6	% fluorescent, day 7	% fluorescent, all days
1	6	337 / 340 (99%)	1766 / 1770 (>99%)	585 / 593 (99%)	52 / 53 (98%)	2716 / 2732 (>99%)
2	8	344 / 344 (100%)	960 / 960 (100%)	617 / 620 (>99%)	Not scored	1921 / 1924 (>99%)

Table S2. Percent of mosquitoes recaptured in BGS2 trap by fluorescent spray treatment. Recaptured and screened mosquitoes had similar counts compared to the number of mosquitoes counted and released before trapping. Some mosquitoes in some replicates escaped trapping. In no replicate were more marked mosquitoes observed from trap collections than originally released (no false positives).

Rep	# marked mosquitoes released	# unmarked mosquitoes released	# marked mosquitoes recaptured	# unmarked mosquitoes recaptured
1	50	51	50 / 50 100%	47 / 51 (92%)
2	27	23	25 / 27 (93%)	22 / 23 (96%)
3	35	24	35 / 35 (100%)	24 / 24 (100%)
4	29	26	29 / 29 (100%)	26 / 26 (100%)
5	32	30	28 / 32 (88%)	30 / 30 (100%)

References

1. Kassambara A, Kosinski M, Biecek P, Fabian S, 2019. R ‘survminer’ package. Available at: https://rpkgs.datanovia.com/survminer/index.html
